# Poor survival is associated with the methylated degree of zinc-finger protein 545 (ZNF545) DNA promoter in gastric cancer

**DOI:** 10.18632/oncotarget.2916

**Published:** 2015-02-26

**Authors:** Jingyu Deng, Han Liang, Guoguang Ying, Qiuping Dong, Rupeng Zhang, Jun Yu, Daiming Fan, Xishan Hao

**Affiliations:** ^1^ Department of Gastroenterology, Tianjin Medical University Cancer Hospital, City Key Laboratory of Tianjin Cancer Center and National Clinical Research Center for Cancer, Tianjin, China; ^2^ Central laboratory, Tianjin Medical University Cancer Hospital, City Key Laboratory of Tianjin Cancer Center and National Clinical Research Center for Cancer, Tianjin, China; ^3^ Institute of Digestive Disease, Li Ka Shing Institute of Health Science, Chinese University of HongKong, Shatin, HongKong; ^4^ State Key Laboratory of Cancer Biology and Institute of Digestive Diseases, Xijing Hospital, Fourth Military Medical University, Xi'an, China

**Keywords:** stomach, neoplasm, zinc-finger protein, survival, methylation

## Abstract

Zinc-finger protein 545 (ZNF545) was identified as a gastric tumour suppressor and potentially independent prognostic factor. At the present study, we found that lower expression of ZNF545 was specific in gastric cancer (GC) tissues, and the inconsistently methylated levels of ZNF545 promoter were identified in the gastric cancer tissues. In the methylation-specific PCR (MSP) analysis cohort, we found that GC patients with hypermethylated ZNF545 promoter exhibited significantly shorter median OS than those with unmethylated ZNF545 promoter and those with hypomethylated ZNF545 promoter. In the other cohort, we also demonstrated that GC patients with three or more methylated CpG sites in the ZNF545 promoter were significantly associated with poor survival by using the bisulphite gene sequencing (BGS). The methylated degrees of five CpG sites (−232, −214, −176, −144 and −116) could also provide distinct survival discrimination of patients with GC. These findings indicated that the methylated CpG sites of the ZNF545 promoter could be used for the clinical prediction of the prognosis of GC.

## INTRODUCTION

Despite great improvements in traditional diagnosis and treatments, gastric cancer (GC) is accounted for 8% of all cancer cases and 10% of all cancer deaths worldwide [1]. GC is highly prevalent in Asia, particularly in China, and considered as one of the leading causes of cancer-related death in the world [1]. As such, detailed molecular mechanisms involved in the development and progression of GC should be identified and characterised to improve patient prognosis. However, tumour development results from multiple genetic alterations. Similar to other types of cancer, GC develops via a multistage process, including DNA methylation of CpG islands, post-translational modification of histones, microRNAs and non-coding RNAs and nucleosome positioning [2–5]. Genetic alterations are considered as key tumourigenic events and may be involved in gastric tumourigenesis [6–11]. Promoter methylation is also considered as one of the major mechanisms to inactivate tumour-related genes, particularly tumour suppressor genes, thereby causing gastric carcinogenesis [12]. Therefore, promoter methylation should be considered as an important hallmark of the initiation and progression of GC.

Zinc-finger proteins (ZFPs), among the most common transcription factors in mammals, constitute the largest family of transcriptional regulators in the human genome [13]. Several ZFPs, such as ZNF217 and ZNF639, are oncogenic proteins [14, 15], whereas other ZFPs, such as ZAC, ST18, ZNF382 and ZNF331, are tumour suppressors [16–19]. In a previous study, ZNF545 was identified as a gastric tumour suppressor and potentially independent prognostic factor in 79 GC cases [20]. In this study, the methylation degrees of ZNF545 promoter were detected in 300 GC tissues by qualitative analysis (methylation-specifi PCR, MSP) and in 158 GC tissues by quantitative analyses (bisulphite gene sequencing, BGS) to determine the precise prognostic prediction values of ZNF545 promoter methylation in patients with GC.

## RESULTS

### Patient demographics

The clinicopathological characteristics of the patients and the methylation status of the ZNF545 promoter are listed in Table 1. The median OS of all patients was 21 months, ranging from 2 months to 95 months. Only 61 (13.32%) patients with GC are alive even after follow up was completed.

**Table 1:** Patient information

Cohort	300 patients with MSP analyisis	158 patients with BGS analysis
Gender		
Male	201 (67.00%)	112 (70.89%)
Female	99 (33.00%)	46 (29.11%)
Age at surgery		
≤ 60	181 (60.33%)	90 (56.96%)
> 60	119 (39.67%)	68 (43.04%)
Tumor size		
< 4.0	39 (13.00%)	27 (17.09%)
≥ 4.0	261 (87.00%)	131 (82.81%)
Tumor location		
Upper third	69 (23.00%)	43 (27.21%)
Middle third	81 (27.00%)	36 (22.78%)
Lower third	131 (43.67%)	71 (44.94%)
More than 2/3 stomach	19 (6.33%)	8 (5.06%)
Depth of tumor invasion (T stage)		
T1	3 (1.00%)	2 (1.27%)
T2	28 (9.33%)	19 (12.03%)
T3	185 (61.67%)	98 (62.02%)
T4	84 (28.00%)	39 (24.68%)
Number of metastatic lymph nodes (N stage)		
N0	80 (26.67%)	31 (19.62%)
N1	105 (35.00%)	57 (36.08%)
N2	64 (21.33%)	43 (27.22%)
N3	51 (17.00%)	27 (17.08%)
Location of lymph node metastasis		
No	80 (26.67%)	31 (19.62%)
Perigastric	95 (31.67%)	63 (39.87%)
Extragastric	125 (41.66%)	64 (40.51%)
Lauren classification		
Intestinal	82 (27.33%)	40 (25.32%)
Diffuse	211 (70.33%)	107 (67.72%)
Mixed	7 (2.34%)	11 (6.96%)
ZNF545 promoter methylation (MSP)		
Hypermethylation	72 (24.00%)	–
Hypomethylation	61 (20.33)	–
Non-methylation	167 (55.67%)	–
Methylated CpG site count (BGS)		
2 or less	–	76 (48.10%)
3 or more	–	82 (51.90%)
Methylated status of CpG −232 (BGS)		
Unmethylated	–	94 (59.49%)
Methylated	–	64 (40.51%)
Methylated status of CpG −214 (BGS)		
Unmethylated	–	84 (53.16%)
Methylated	–	74 (46.84%)
Methylated status of CpG −176 (BGS)		
Unmethylated	–	86 (54.43%)
Methylated	–	72 (45.57%)
Methylated status of CpG −144 (BGS)		
Unmethylated	–	86 (54.43%)
Methylated	–	72 (45.57%)
Methylated status of CpG −116 (BGS)		
Unmethylated	–	96 (60.76%)
Methylated	–	62 (39.24%)

### Protein and mRNA expressions of ZNF545 in 25 GC tissues and 25 normal gastric mucosal tissues

**Figure 1:**
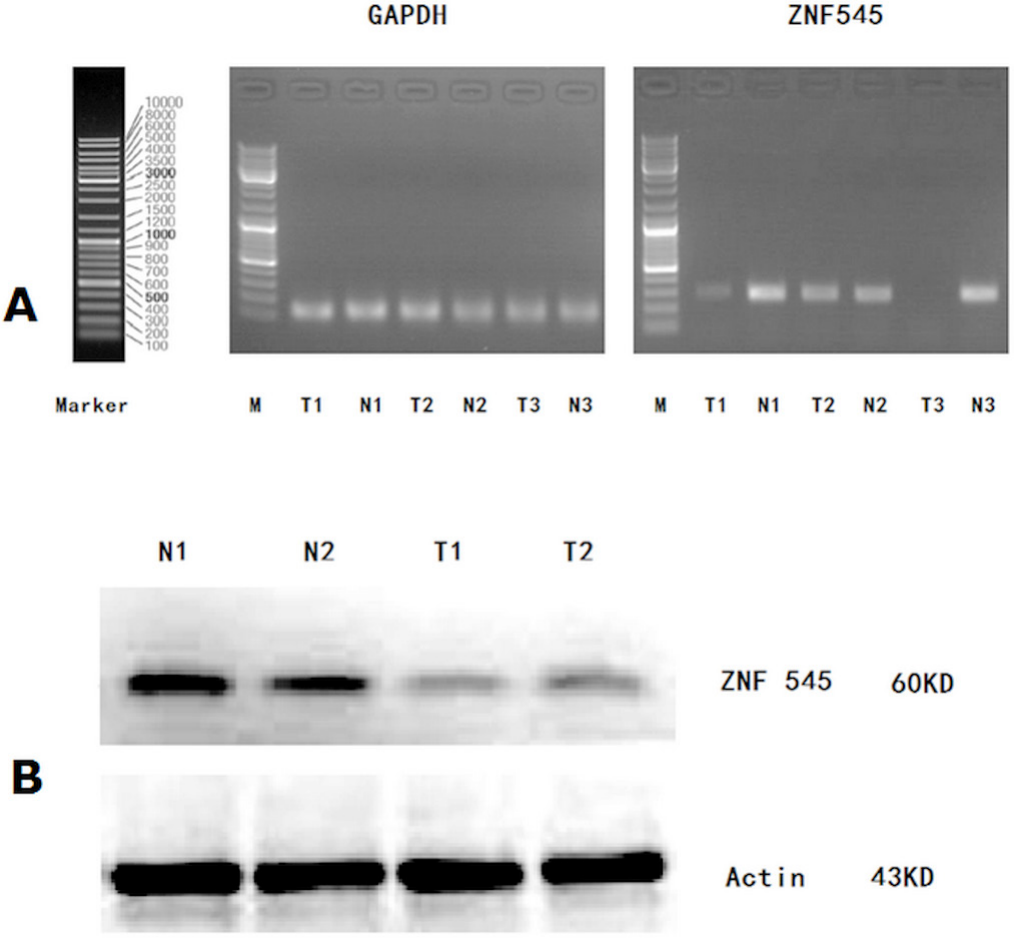
**(A)** ZNF545 mRNA expression (RT-PCR) in 25 GC tissues and that in 25 normal gastric mucosal tissues; **(B)** Western Blot analysis for ZNF545 protein expression in 25 GC tissues and that in 25 normal gastric mucosal tissues. (Representation: T, GC tissues; N: normal gastric mucosal tissues).

The mRNA expression of ZNF545 was detected in 25 of the 158 GC tissues and the 25 normal gastric mucosal tissues by RT-PCR (Figure 1A). The relative mRNA expression of ZNF545 in the 25 GC tissues was significantly lower than that in the 25 normal gastric mucosal tissues (0.321 ± 0.108 vs. 1.107 ± 0.315, *P* < 0.001). The protein expression of ZNF545 was also simultaneously detected in 25 of the 158 GC tissues and the 25 normal gastric mucosal tissues by western blot (Figure 1B). The relative protein expression value of ZNF545 in the 25 GC tissues was significantly lower than that in the 25 normal gastric mucosal tissues (0.482 ± 0.116 vs. 1.468 ± 0.250, *P* = 0.009).

### Hypermethylation of the ZNF545 promoter in 300 GC tissues

**Figure 2:**
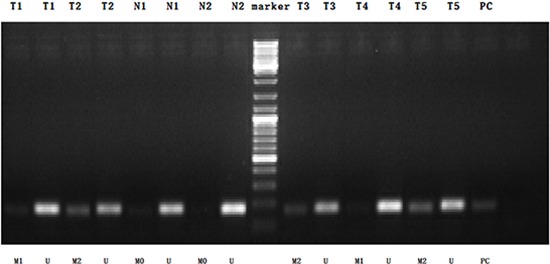
MSP detection of ZNF545 promoter methylation in different GC tissues and normal gastric mucosal tissues (Representation: T, GC tissues; N, normal gastric mucosal tissues; M0, non-methylation; M1, hypomethylation; M2, hypermethylation; U, unmethylation).

We conducted MSP analysis and detected the different degrees of methylation (including hypermethylation, hypomethylation and non-methylation) of the ZNF545 promoter in 300 GC tissues; by contrast, the ZNF545 promoter was not methylated in the 25 normal gastric mucosal tissues (Figure 2). Among the 300 GC tissues, 72 (24%) presented hypermethylated ZNF545 promoter, 61 (20.33%) revealed hypomethylated ZNF545 promoter and 167 (55.67%) presented with unmethylated ZNF545 promoter.

### Methylated CpG sites of the ZNF545 promoter in 158 GC tissues

**Figure 3:**
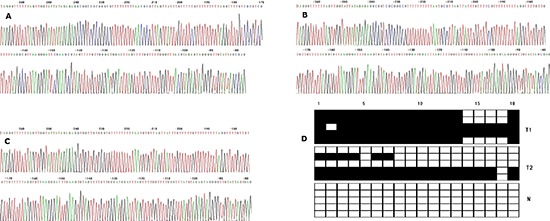
**(A)** Bisulphite sequencing figure of ZNF545 in GC tissue 1, **(B)** Bisulphite sequencing figure of ZNF545 in GC tissue 2, **(C)** Bisulphite sequencing figure of ZNF545 in normal gastric mucosal tissue, and **(D)** Bisulfite sequencing results in GC tissues and in normal gastric mucosal tissue. (Representation: T, GC tissues; N: normal gastric mucosal tissues).

The methylated CpG site count of the 158 patients with GC ranged from 0 to 18 with an average methylated CpG site count of 8.06. Among the 158 patients included in the study, 119 (75.32%) presented one or more methylated CpG sites and 39 (24.68%) showed no methylated CpG sites. The patients without methylated CpG sites exhibited higher median OS than those with one or more methylated CpG sites (27 months vs. 20 months), but the median OS was not statistically different between the two groups of patients (*P* = 0.679). The result of the cut-point analysis of the methylated CpG site count showed that 82 patients (51.90%) exhibited three or more methylated CpG sites and 76 patients (48.10%) presented two or less methylated CpG sites. No methylated CpG sites were found in the normal gastric mucosal epithelial tissues. The images showing the methylated sequences and the charts of CpG site are shown in Figure 3.

### Survival analysis

**Figure 4:**
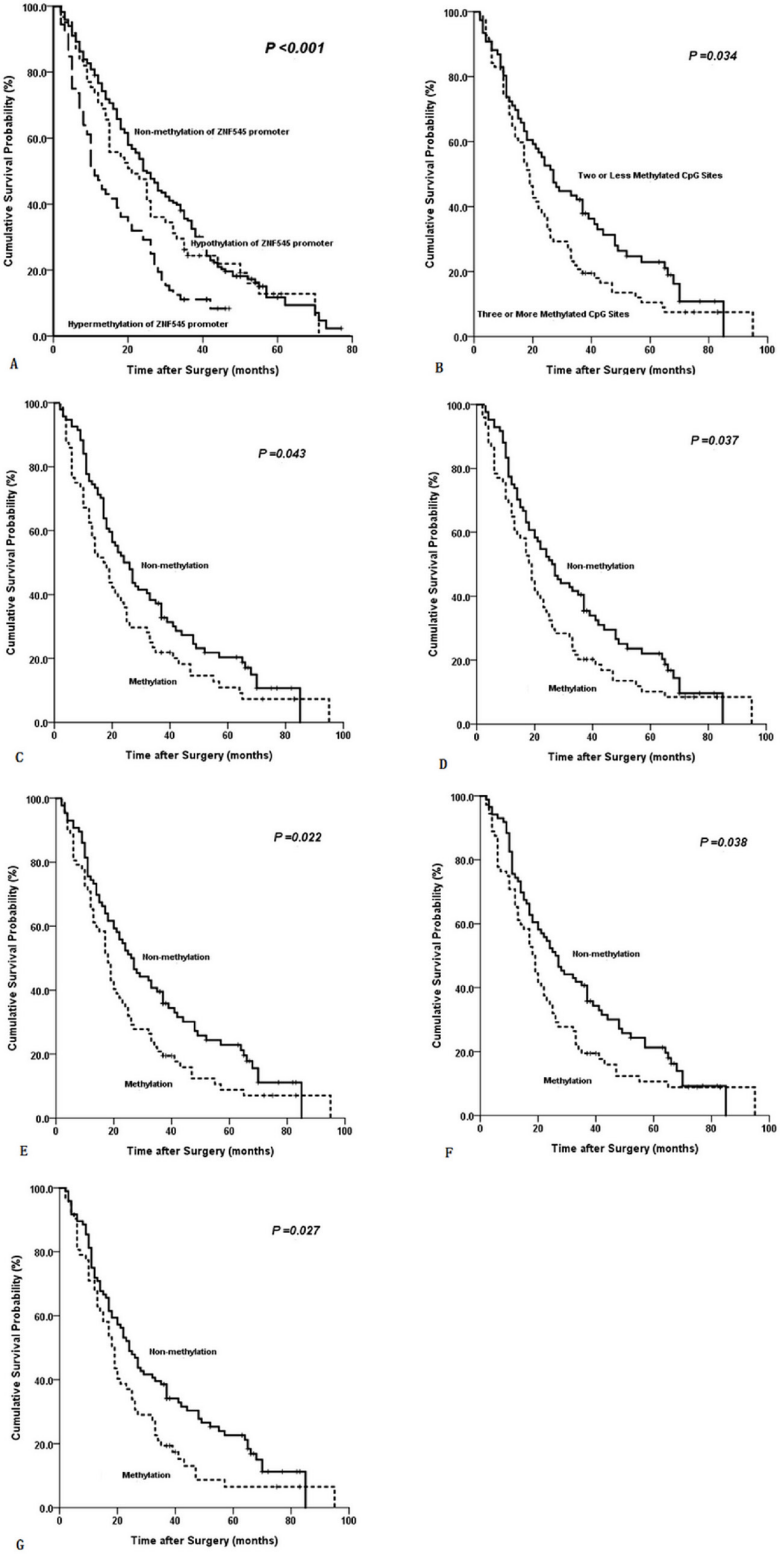
**Kaplan-Meier survival curves comparing months of survival in gastric cancer patients are shown for (A)** methylated statuses of ZNF545 promoter (MSP), **(B)** methylated CpG site count of ZNF545 promoter, **(C)** methylated status of CpG −232, **(D)** methylated status of CpG −214, **(E)** methylated status of CpG −176, **(F)** methylated status of CpG −144, and **(G)** methylated status of CpG −116.

In univariate survival analysis, four clinicopathological characteristics were significantly associated with the survival of 300 patients with GC. In MSP analysis, methylated ZNF545 promoter was observed in the same patients. The following characteristics were observed (Table 2): T stage (*P* < 0.001); N stage (*P* < 0.001); location of lymph node metastasis (*P* < 0.001); and gender (*P* = 0.048). Although the methylation of the ZNF545 promoter was significantly associated with the OS of patients, as indicated in Kaplan-Meier curves (*P* < 0.001; Table 2; Figure 4), no statistical survival difference was found between 61 patients with hypomethylated ZNF545 promoter and 167 patients with unmethylated ZNF545 promoter (*P* = 0.373). These factors were included in a multivariate Cox proportional hazard model to adjust the effects of covariates. N stage (HR = 1.520, *P* < 0.001) and T stage (HR = 1.432, *P* = 0.001) were identified as the independent predictors of the OS of patients with GC rather than ZNF545 promoter methylation, as revealed by the multivariate survival analysis (Table 2).

**Table 2:** Survival analysis of 300 GC patients with MSP detection of ZNF545 promoter methylation

Variables	Median OS (mo)	*X*^2^ value	Univariate *P* value	HR value	Multivariate *P* value
Gender					
Male	23	3.922	0.048		
Female	17				
Age at surgery (years)					
≤ 60	19	0.193	0.661		
> 60	24				
Tumor location					
Upper third	22	1.846	0.605		
Middle third	16				
Lower third	24				
≥ 2/3 stomach	12				
Tumor size (cm)					
< 4.0	26	1.054	0.305		
≥ 4.0	21				
Lauren classification					
Intestinal	24	3.513	0.173		
Diffuse	20				
Mixed	24				
Depth of tumor invasion (T stage)					
T1	46	21.861	< 0.001	1.432 (1.159–1.769)	0.001
T2	25				
T3	24				
T4	14				
Number of metastatic lymph nodes (N stage)					
N0	34	59.155	< 0.001	1.520 (1.343–1.721)	< 0.001
N1	23				
N2	17				
N3	9				
Location of lymph node metastasis					
No	34	27.942	< 0.001		
Perigastric	20				
Extragastric	15				
ZNF545 promoter methylation					
Hypermethylation	11	20.267	< 0.001		
Hypomethylation	21				
Non-methylation	25				

In univariate survival analysis, four clinicopathological characteristics were significantly associated with the survival of 158 patients with GC and with methylated ZNF545 promoter (as shown in BGS analysis). The following characteristics were observed (Table 3): T stage (*P* < 0.001); N stage (*P* < 0.001); location of lymph node metastasis (*P* < 0.001); and tumour location (*P* = 0.022). The methylated CpG site count (*P* = 0.034), the methylated status of CpG −232 (*P* = 0.043), the methylated status of CpG −214 (*P* = 0.037), the methylated status of CpG −176 (*P* = 0.022), the methylated status of CpG −144 (*P* = 0.038) and the methylated status of CpG −116 (*P* = 0.027) were significantly associated with the OS of patients, as shown in Kaplan-Meier curve discrimination (Table 1; Figure 4). These 10 factors were included in a multivariate Cox proportional hazard model to adjust the effects of covariates. In multivariate analysis, the methylated status of CpG −232 (HR = 1.490, *P* = 0.023), N stage (HR = 1.659, *P* < 0.001) and T stage (HR = 1.659, *P* = 0.001) were identified as the independent predictors of the OS of patients with GC after surgery (Table 3). In addition, T stage yielded a smaller BIC value than N stage and the methylated status of CpG −232, representing the optimal prognostic predictor of GC (Table 3).

**Table 3:** Survival analysis of 158 GC patients with BGS detection of ZNF545 promoter methylation

Variables	Median OS (mo)	*X*^2^ value	Univariate *P* value	HR value	Multivariate *P* value	BIC value
Gender						
Male	20	0.911	0.340			
Female	22					
Age at surgery (years)						
≤ 60	20	0.011	0.915			
> 60	21					
Tumor location						
Upper third	17	9.642	0.022			
Middle third	20					
Lower third	26					
≥ 2/3 stomach	16					
Tumor size (cm)						
< 4.0	27	3.040	0.081			
≥ 4.0	20					
Lauren classification						
Intestinal	26	3.430	0.180			
Diffuse	20					
Mixed	17					
Depth of tumor invasion (T stage)						
T1	70	20.677	< 0.001	1.659 (1.224–2.249)	0.001	56.968
T2	33					
T3	23					
T4	12					
Number of metastatic lymph nodes (N stage)						
N0	48	43.150	< 0.001	1.659 (1.381–1.993)	< 0.001	74.211
N1	25					
N2	18					
N3	11					
Location of lymph node metastasis						
No	48	23.436	< 0.001			
Perigastric	17					
Extragastric	18					
Methylated CpG site count						
2 or less	27	4.497	0.034			
3 or more	19					
Methylated status of CpG −232						
Unmethylated	24	4.109	0.043	1.490 (1.057–2.099)	0.023	68.817
Methylated	17					
Methylated status of CpG −214						
Unmethylated	26	4.353	0.037			
Methylated	18					
Methylated status of CpG −176						
Unmethylated	26	5.250	0.022			
Methylated	18					
Methylated status of CpG −144						
Unmethylated	26	4.321	0.038			
Methylated	18					
Methylated status of CpG −116						
Unmethylated	24	4.884	0.027			
Methylated	18					

### Correlation analysis

Table 4 shows the correlation analysis results between the methylated status of the CpG sites of the ZNF545 promoter and patient demographics. The methylated status of four CpG sites (−214, −176, −144 and −116) was significantly correlated with the age of the patients with GC subjected to surgery (*P* = 0.048, *P* = 0.010, *P* = 0.024 and *P* = 0.002). The methylated status of two CpG sites (−232 and −144) was significantly correlated with the tumour sizes of 158 patients with GC (*P* = 0.011 and *P* = 0.024). The methylated status of two CpG sites (−214 and −176) were also correlated with Lauren classification of primary tumour (*P* = 0.026 and *P* = 0.016).

**Table 4:** Correlation analysis between methylated status of CpG sites of ZNF545 promoter and clinicopathological characteristics

Variables	Methylated CpG sites ≥ 3	Methylated CpG −232	Methylated CpG −214	Methylated CpG −176	Methylated CpG −144	Methylated CpG −116
Gender						
Male	60	48	53	54	51	46
Female	22	16	21	18	21	16
*P* value	0.511	0.348	0.849	0.298	0.989	0.462
Age at surgery (years)						
≤ 60	42	31	36	33	34	26
> 60	40	33	38	39	38	36
*P* value	0.130	0.074	0.048	0.010	0.024	0.002
Tumor location						
Upper third	25	23	21	22	23	18
Middle third	18	14	19	17	16	14
Lower third	35	25	31	30	29	27
≥ 2/3 stomach	4	2	3	3	4	3
*P* value	0.819	0.198	0.766	0.775	0.613	0.981
Tumor size (cm)						
< 4.0	10	5	9	9	7	8
≥ 4.0	72	59	65	63	65	54
*P* value	0.090	0.011	0.123	0.161	0.024	0.261
Lauren classification						
Intestinal	23	19	21	21	19	15
Diffuse	50	39	44	42	45	40
Mixed	9	6	9	9	8	7
*P* value	0.061	0.295	0.026	0.016	0.145	0.229
Depth of tumor invasion (T stage)						
T1	0	0	0	0	0	0
T2	9	8	9	8	9	8
T3	51	39	45	44	42	38
T4	22	17	20	20	21	16
*P* value	0.452	0.669	0.551	0.513	0.381	0.701
Number of metastatic lymph nodes (N stage)						
N0	16	12	14	14	15	11
N1	30	24	28	26	24	22
N2	17	17	17	17	17	15
N3	19	11	15	15	16	14
*P* value	0.096	0.989	0.594	0.633	0.385	0.505
Location of lymph node metastasis						
No	16	12	14	14	15	11
Perigastric	31	22	28	27	28	25
Extragastric	35	30	32	31	29	26
*P* value	0.826	0.380	0.804	0.818	0.936	0.887

## DISCUSSION

The prognosis of GC is mainly dependent on clinical stage at diagnosis and treatment [21]. DNA methylation is a major mechanism by which tumour suppressor genes are inactivated in many kinds of cancer cells [22]. In human cancers, some ZFPs, including ZAC, ST18, ZNF382 and ZNF331, function as tumour suppressors and are frequently inactivated by CpG methylation [16–19]. However, other ZFPs are potential oncogenic activators [14, 15]. Tumour suppressor genes involved in cell proliferation are aberrantly inactivated by promoter methylation; this inactivation is frequently involved in multiple processes of tumourigenesis [23]. ZNF545, as a tumour suppressor gene, is potentially downregulated by promoter methylation in multiple tumours, including 13 GC tissues [24]. Cheng et al [24] demonstrated that ZNF545 can repress NF-kB and AP-1 signaling pathways, whereas ectopic ZNF545 expression in silenced tumour cells significantly inhibits tumour growth and induces apoptosis. Functional studies have shown that ZNF545 is involved in ribosome biogenesis by inhibiting rDNA promoter activity and decreasing cellular protein translation efficiency [24]. These findings have indicated that the tumour-specific methylation of ZNF545 can be performed as an epigenetic biomarker to diagnose cancer [24].

In a previous study, ZNF545 participates as a functional tumour suppressor of GC by inhibiting rRNA transcription; the methylation of ZNF545 is also considered as an independent prognostic factor of 79 patients with GC [20]. Furthermore, ZNF545 is silenced or reduced in GC cell lines and tissues because the promoter was methylated, as revealed by MSP and BGS detection. The ZNF545 promoter is also methylated in 51.9% of the GC tissues but not in normal gastric mucosal tissues [20]; this result is similar to that in the present study. In the qualitative analysis of methylation, methylated ZNF545 may be regarded as a valuable new prognostic factor of patients with early stage GC [20]. However, we proposed that an optimal prognostic predictor should be detected in patients suffering from all stages of cancer. We detected the mRNA and protein of ZNF545 gene expression in 25 GC tissues and 25 normal gastric mucosal tissues; we also detected significantly lower expression of ZNF545 in GC tissues than in normal gastric mucosal tissues. The methylated ZNF545 promoter was qualitatively detected in 300 GC tissues by conducting MSP analysis. We found that the survival rate of patients with hypomethylated ZNF545 promoter was not significantly different from that of patients with unmethylated ZNF545 promoter; the patients with hypermethylated ZNF545 promoter exhibited shorter OS than the other patients (*P* < 0.001). Therefore, only the hypermethylation of the ZNF545 promoter was significantly associated with the prognostic prediction of GC.

We performed BGS analysis and quantitatively detected the methylation of ZNF545 promoter in other 158 GC tissues and 25 normal gastric mucosal tissues, including advanced stage GC tissues, to demonstrate the prognostic prediction value of ZNF promoter methylation. Approximately 75.32% (119/158) of the GC tissues contained one or more methylated CpG sites and none of these sites were found in normal gastric mucosal tissues; this result may indicate that the methylation of ZNF545 promoter is GC specific. We initially failed to demonstrate the statistically prognostic differences between 119 patients with GC and ZNF545 promoter methylation and 39 patients with GC but without ZNF545 promoter methylation. However, differentially methylated CpG site detection has been used to evaluate the correlation between the methylated levels of gene promoter and various abnormally biological events [25–28]. Therefore, we decided to meticulously analyse the methylated CpG sites of ZNF545 promoter in 158 patients by using the comparatively quantitative BSP method with no less than five clones of each GC sample [29, 30]. We then demonstrated that patients with GC and with three or more methylated CpG sites exhibited significantly shorter survival than those with two or less methylated CpG sites of ZNF545 promoter; this result is consistent with that of the cut-point survival analysis. Five methylated CpG sites (−232, −214, −176, −144 and −116) of the ZNF545 promoter were associated with the OS of 158 patients with GC. Hence, these findings provided relevant information that could be used to further investigate the diagnosis, intervention and prognosis of GC. In multivariate survival analysis (Cox regression with the forward step procedures), the methylated status of CpG −232 of the ZNF545 promoter was considered as an independent prognostic predictor of 158 patients with GC, indicating the importance of ZNF545 promoter methylation.

In contrast to the results of the multivariate survival analysis of 300 patients with GC by conducting MSP detection, our finding of the methylated status of CpG −232 exhibited a smaller BIC value than that of N stage. This result indicated that this methylated status was the most effective potential prognostic predictor of GC. We also found that the methylated CpG sites of the ZNF545 promoter were associated with older, larger and diffuse type of GC based on Lauren classification. These clinicopathological characteristics related to the methylated CpG sites of the ZNF545 promoter correspond to the biological behaviours of GC cells, which were discovered in previous studies [20, 24].

## PATIENTS AND METHODS

### Data source

After approval from the Tianjin Medical University Cancer Hospital institutional review board, data from the cancer registry of the Tianjin Cancer Institute were obtained. Oral and written informed consents were also obtained from patients who were included in this study. Data obtained from the registry were listed as follows: age, gender, tumour location, tumour size, depth of tumour invasion (T stage, according to the Sixth Edition of UICC TNM Classification for GC), number of metastatic lymph nodes (N stage, according to the Sixth Edition of UICC TNM Classification for GC), extent of lymph node metastasis, Lauren classification, and follow-up vital status.

### Patients and study samples

To analyse ZNF545 promoter methylation, we collected 458 fresh GC tissues from patients with GC and who underwent curative gastrectomy between April 2003 and December 2007 at the Department of Gastroenterology, Tianjin Medical University Cancer Hospital. A cohort of 25 normal gastric mucosal epithelial tissues from normal people was also obtained between 2004 and 2007 at the Department of Endoscopic Examination and Treatment, Tianjin Medical University Cancer Hospital. The tumour and normal gastric mucosal epithelial tissue samples were histologically verified. The patients were not subjected to radiation, chemical or biological treatment before potentially curative gastrectomy was performed. Adjuvant chemotherapy or radiotherapy was not routinely administered to the patients. The clinicopathological characteristics of the two cohorts are summarised in Table 1. Consent regarding the use of tissue samples and records was obtained from each patient. The Institutional Research Ethics Committee of Tianjin Medical University Cancer Hospital approved the study protocol and provided permission to use patient clinical data.

### Surgical treatment

Curative resection was defined as the complete absence of grossly visible tumour tissues and metastatic lymph nodes remaining after resection was performed with pathologically negative resection margins. Primary tumours were resected en bloc with limited or extended lymphadenectomy (D1 or D2–3 according to the Japanese Gastric Cancer Association). Surgical specimens were evaluated according to the Sixth UICC TNM Classification for GC.

### DNA and RNA extraction

Genomic DNA was extracted from the 458 GC tissues and the 25 normal gastric mucosal tissues by using a QIAamp DNA mini kit (Qiagen, Valencia, CA) according to the manufacturer's instructions. Genomic DNA was modified using sodium bisulphite in EZ DNA Methylation-Gold^TM^ kit (Zymo Research, Hornby, Canada). RNA was extracted from 25 of the 458 GC tissues and 25 normal gastric mucosal tissues by using Trizol reagent (Invitrogen, Carlsbad, CA) according to the manufacturer's instructions.

### Western blot and semi-quantitative reverse transcription PCR (RT-PCR)

A total of 25 of the 458 GC tissues and 25 normal gastric mucosal tissues were each added to 1 mL of 100 mmol/L Tris/HCl (pH 7.5), 100 mmol/L NaCl, 0.5% sodium deoxycholate, 1 mmol/L ethylenediaminetetraacetic acid, 1% Nonidet P-40, 0.1% sodium dodecyl sulphate and protease inhibitor. After blocking was performed, 50 μg of the sample was incubated for 60 min with rabbit anti-ZNF545 (Santa Cruz, sc-102235, 1:500 dilution) at room temperature. A gel imager system (Asia Xingtai Mechanical and Electrical Equipment Company, Beijing, China) was used to analyse images and determine gray values.

The mRNA expression of ZNF545 was detected by subjecting 25 of the 458 GC tissues and the 25 normal gastric mucosal tissues to RT-PCR. Total RNA was reverse-transcribed to cDNA in a 20 μl solution by using a reverse transcription kit (Invitrogen, Carlsbad, CA). The primers designed and utilised for ZNF545 were listed as follows: forward sequence 5ʹ-TAAACCCCAAGGAGGACTGAC-3ʹ and reverse sequence 5ʹ-TCCAACATGACATCTCTGTACAAGT-3ʹ. The GAPDH gene was used as an endogenous control of semi-quantitative DNA-PCR. Primers designed and utilised for GAPDH were listed as follows: forward sequence 5ʹ-GAAGGTGAAGGTCGGAGTC-3ʹ and reverse sequence 5ʹ-GAAGATGGTGATGGGATTTC-3ʹ. The following PCR cycling conditions were applied: 35 cycles of denaturation at 95°C for 3 min; annealing at 94°C for 30 s; and extension at 56°C for 30 s; and a final extension at 72°C for 8 min. PCR products were electrophoresed on 2% agarose gel with ethidium bromide and visualised using a gel imager system (Asia Xingtai Mechanical and Electrical Equipment Company, Beijing, China).

### Methylation-specific PCR (MSP)

A total of 300 GC tissues and 25 normal gastric mucosal tissues were subjected to qualitative methylation analysis of the ZNF545 promoter by methylation-specific PCR (MSP). The following ZNF545 primers were used to detect the methylated (M) or unmethylated (U) alleles of the ZNF5 promoter: for methylated alleles, ZNF545-MF 5ʹ-GGTATTATAGAGAGGCGGTCGC-3ʹ and ZNF545-MR 5ʹ-ACTCTACGTAAACCCGAAACCG-3ʹ; for unmethylated alleles, ZNF545-UF 5ʹ-AGTT GGTATTATAGAGAGGTGGTTGTG-3ʹ and ZNF545-UR 5ʹ-CCTACTCTACATAAACCCAAAACCA-3ʹ. A total of 25 cycles of MSP were performed using Ampli *Taq*-Gold (methylation-specific primers, annealing temperature 600°C; unmethylation-specific primers, annealing temperature 580°C). MSP primers were initially evaluated to verify whether or not any unbisulphited DNA is amplified, and the specificity of MSP was further confirmed by directly sequencing some PCR products. PCR was resolved using 2% agarose gel. Image J software was used to calculate the relative values of the methylation of the ZNF545 promoter in the 300 GC tissues. The hypermethylation of the ZNF545 promoter was defined as the calculated methylated value of GC tissue but not less than the positive control value.

### Bisulphite genomic sequencing (BGS)

The methylation of the ZNF545 promoter in 158 GC tissues and 25 normal gastric mucosal tissues was qualitatively analysed by BGS. The genomic DNA was modified using sodium bisulphite in an EZ DNA Methylation-Gold™ kit (Zymo Research, Hornby, Canada). Hot start PCR with bisulphite-treated DNA was performed using a 192 bp PCR product spanning the promoter region from −268 bp to −77 bp relative to the transcription start site of ZNF545. The promoter region of ZNF545 contains 18 CpG sites. The sequences of the PCR primers were listed as follows: F5ʹ-TAGGGTTTTTTAGTTGGTATTATAGAGAG-3ʹ and R5ʹ-CTCRCTAATACAACCCCTACTCTAC-3ʹ. The purified PCR products were cloned into the pUC18-T vector (Biodee, Beijing, China), and at least five clones from each sample were randomly selected and sequenced by Shanghai Sangon Co. (Shanghai, China).

### Follow up

After curative surgery, all of the patients were followed up every three months or six months for two years at the outpatient department; these patients were also followed up every year from the third year to the fifth year. Thereafter, these patients were followed up annually until their death. The median follow up of the entire cohort was 41 months, ranging from 1 month to 104 months. The follow up of the patients included in this study was completed in December 2012. Ultrasonography, CT scans, chest X-ray and endoscopy were performed in every visit.

### Statistical analysis

Median overall survival (OS) was determined using Kaplan-Meier method, and log-rank test was performed to determine significance. Potentially important factors in univariate analyses (*P* < 0.05) were included in multivariate analyses. OS was subjected to multivariate analysis by using Cox proportional hazard model with forward step procedures. Hazard ratios (HR) and 95% confidence interval were calculated. Bayesian information criterion (BIC) in a Cox proportional hazard regression model was calculated in terms of different categories to quantify their discriminatory ability. A small BIC value corresponds to an efficient model to predict outcomes. With cut-point survival analysis, the optimal cutoff of the CpG site count was 3. Significance was set at *P* < 0.05. Statistical analyses were performed using SPSS 18.0.
